# Supplementary material for: Receptor-Binding Domain (RBD) Antibodies Contribute More to SARS-CoV-2 Neutralization When Target Cells Express High Levels of ACE2
Source: Viruses. 2022 Sep 16;14(9):2061. doi: 10.3390/v14092061 (PMC9504593; doi:10.3390/v14092061)
Supplement: Supplementary file 1 [file viruses-14-02061-s001.zip › viruses-1914965-supplementary.pdf]

S1

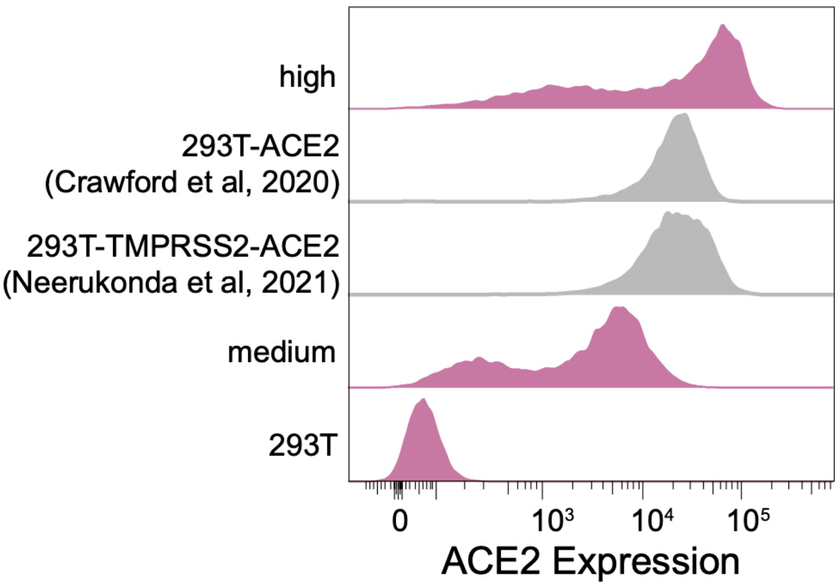

Figure S1. ACE2 expression in previously published ACE2 over-expressing 293T cells commonly used for spike-pseudotyped lentiviral neutralization assays. This figure compares ACE2 expression in the high and medium ACE2 cells described in the current paper (Fig 1A) to the 293T-ACE2 cells described by [14]) and 293T-TMPRSS2-ACE2 cells described by [15]). These data were acquired in a separate experiment from that shown in Figure 1A. The medium and high ACE2 cell clones were not pre-treated with puromycin to purge cells that had lost ACE2 expression prior to running this experiment, likely explaining the larger tail of non-expressing cells relative to Figure 1A.

S2

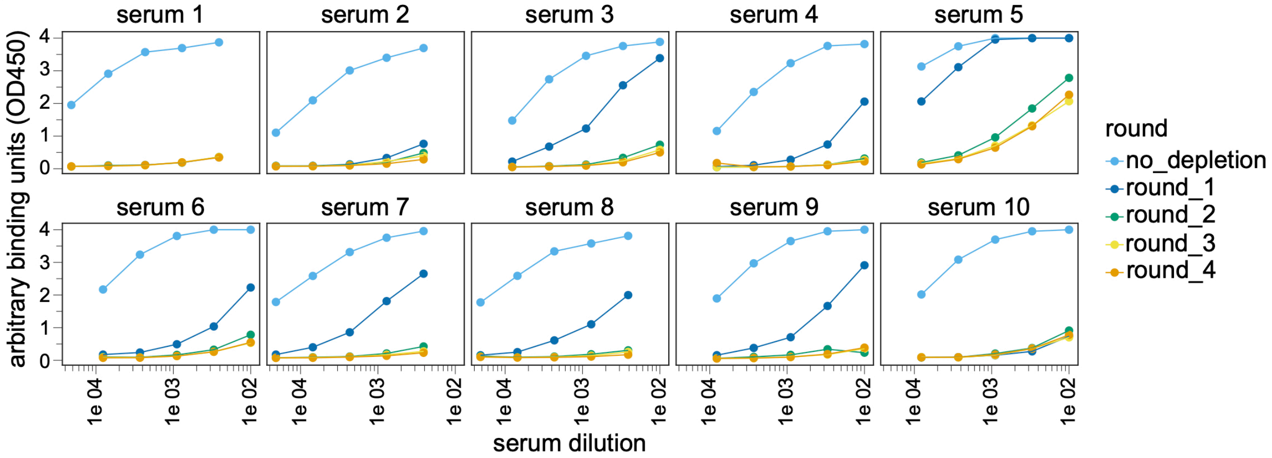

Figure S2. ELISAs showing depletion of RBD-targeting antibodies from sera. ELISA binding curves for serum samples used in lentiviral pseudotype neutralization assays shown in Fig 2. Sera were depleted of RBD-targeting antibodies using four rounds of the process shown in Fig 2A, and binding to RBD was measured by ELISA after each round.

S3

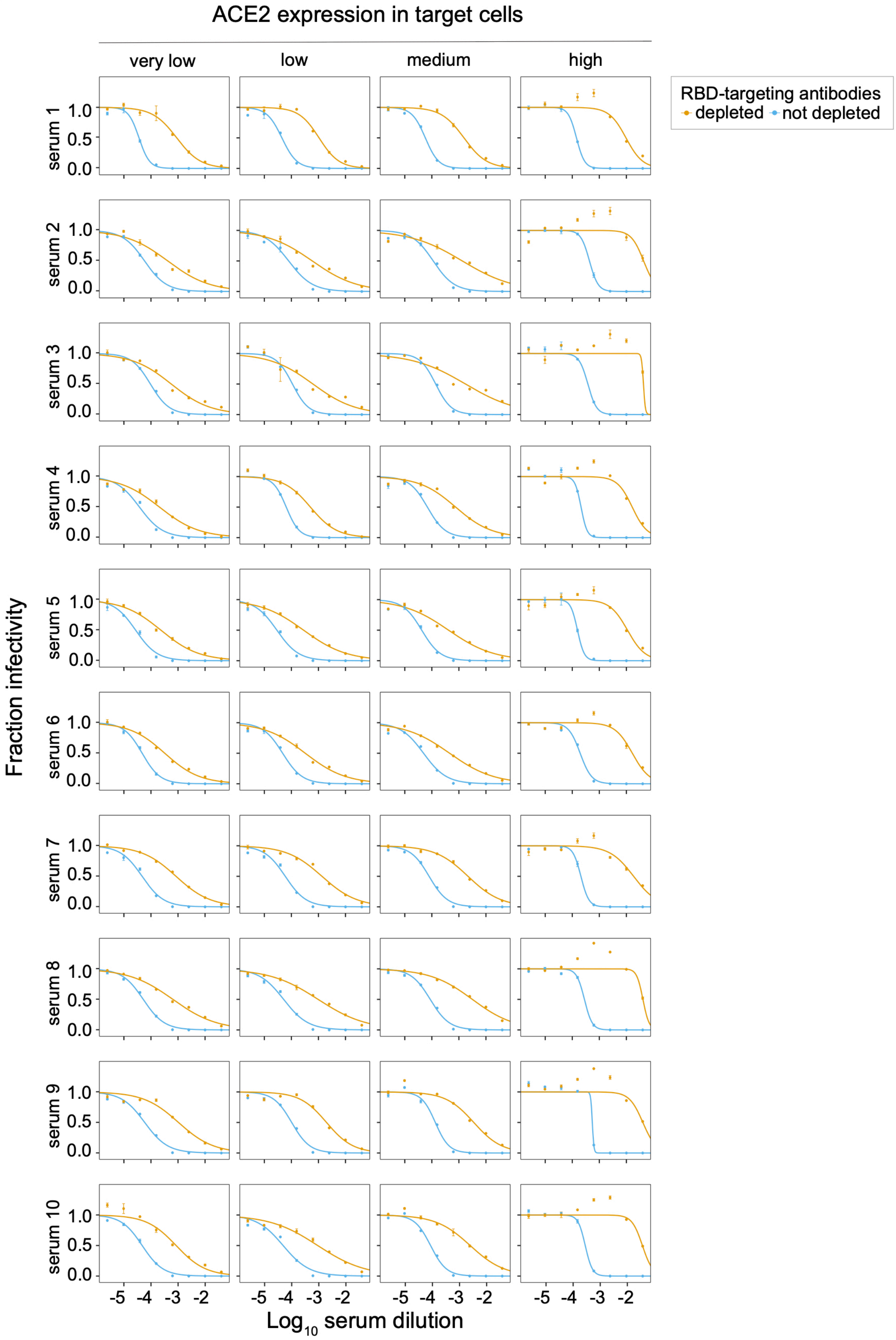

Figure S3. Spike-pseudotyped lentiviral particle neutralization with RBD-depleted and non-depleted sera summarized in Fig 2. Pseudovirus neutralization for RBD-depleted and non-depleted sera was measured on cells expressing different amounts of ACE2. IC_{50} values were calculated using the neutcurve software package (https://jbloomlab.github.io/neutcurve/, version 0.5.7), by fitting a Hill curve and fixing the top of the curve to one and bottom to zero. NT_{50} values from these neutralization curves were used to plot Fig 2B and calculate values for Figures 2C and D.

Table S1. Characteristics of sera used in this study. Participant SARS-CoV-2 immune history, age, sex, illness severity, vaccine type, approximate sample collection date, days post second vaccine dose (at time of sample collection), and approximate date of symptom onset.

Sample	SARS-CoV-2 immune history	Age	Sex	Illness severity	Vaccine	Sample collection date	Days post second dose	Symptom onset
Serum 1	Vaccinated 2x, previously infected	36	Female	Mild, non-hospitalized	Pfizer	Jan-Feb 2021	10	Mar 2020
Serum 2	Vaccinated 2x, previously infected	72	Female	Mild, non-hospitalized	Pfizer	Mar-Apr 2021	15	Mar 2020
Serum 3	Vaccinated 2x, previously infected	50	Male	Mild, non-hospitalized	Pfizer	May-Jun 2021	27	Mar 2020
Serum 4	Vaccinated 2x, previously infected	64	Female	Mild, non-hospitalized	Pfizer	May-Jun 2021	18	Mar 2020
Serum 5	Vaccinated 2x, previously infected	47	Female	Mild, non-hospitalized	Pfizer	Jan-Feb 2021	10	Mar 2020
Serum 6	Vaccinated 2x, previously infected	37	Female	Mild, non-hospitalized	Moderna	May-Jun 2021	36	Apr 2020
Serum 7	Vaccinated 2x, previously infected	60	Female	Mild, non-hospitalized	Pfizer	Mar-Apr 2021	9	Mar 2020
Serum 8	Vaccinated 2x, previously infected	53	Male	Mild, non-hospitalized	Pfizer	Jan-Feb 2021	8	Mar 2020
Serum 9	Vaccinated 2x, previously infected	43	Male	Mild, non-hospitalized	Pfizer	May-Jun 2021	19	Mar 2020
Serum 10	Vaccinated 2x, previously infected	41	Female	Mild, non-hospitalized	Pfizer	May-Jun 2021	29	Jun 2020
